# Collective cell migration is spatiotemporally regulated during mammary epithelial bifurcation

**DOI:** 10.1242/jcs.259275

**Published:** 2023-01-05

**Authors:** Neil M. Neumann, Daniel M. Kim, Robert J. Huebner, Andrew J. Ewald

**Affiliations:** Department of Cell Biology and Center for Cell Dynamics, School of Medicine, Johns Hopkins University, Baltimore, MD 21205, USA

**Keywords:** Collective cell migration, Bifurcation, Branching morphogenesis, Epithelial development, Mammary gland, TGF-β

## Abstract

Branched epithelial networks are generated through an iterative process of elongation and bifurcation. We sought to understand bifurcation of the mammary epithelium. To visualize this process, we utilized three-dimensional (3D) organotypic culture and time-lapse confocal microscopy. We tracked cell migration during bifurcation and observed local reductions in cell speed at the nascent bifurcation cleft. This effect was proximity dependent, as individual cells approaching the cleft reduced speed, whereas cells exiting the cleft increased speed. As the cells slow down, they orient both migration and protrusions towards the nascent cleft, while cells in the adjacent branches orient towards the elongating tips. We next tested the hypothesis that TGF-β signaling controls mammary branching by regulating cell migration. We first validated that addition of TGF-β1 (TGFB1) protein increased cleft number, whereas inhibition of TGF-β signaling reduced cleft number. Then, consistent with our hypothesis, we observed that pharmacological inhibition of TGF-β1 signaling acutely decreased epithelial migration speed. Our data suggest a model for mammary epithelial bifurcation in which TGF-β signaling regulates cell migration to determine the local sites of bifurcation and the global pattern of the tubular network.

## Teaser

**Highlighted Article:** Bifurcation of mammary tubes is accomplished through spatiotemporal regulation of luminal epithelial cell migration and is regulated by TGF-β signaling.

## INTRODUCTION

The mammary ductal network originates as an epithelial placode that invades the underlying mesenchyme, birfurcates, polarizes into a bilayered structure and then pauses until the onset of steroid hormones at the start of puberty (Hogg et al., 1983). During puberty, these polarized ducts proliferate to form a multilayered, low-polarity structure, referred to as a terminal end bud (TEB), that then accomplishes the majority of ductal elongation (Ewald et al., 2008; Hinck and Silberstein, 2005; Paine and Lewis, 2017).

At the molecular level, ductal elongation is governed by a complex interplay between different steroid hormone and receptor tyrosine kinase (RTK) receptor signals that are exchanged between the epithelial and stromal compartments (Hennighausen and Robinson, 2005; McNally and Martin, 2011). At the cellular level, there is an asymmetric cell division that drives the transition from single- to multi-layered architecture in the TEB (Huebner et al., 2014) and an RTK-regulated collective cell migration that drives elongation of the epithelial tubes (Ewald et al., 2008; Huebner et al., 2016; Neumann et al., 2018). This cell migration is characterized by a polarization of protrusions and migration in the direction of tissue elongation, with intercalation between cell layers used as a mechanism both of increasing tube surface area and of resolving the TEB back to a bilayered, polarized tube (Huebner et al., 2016; Neumann et al., 2018).

Epithelial bifurcation is required to elaborate a ductal network, yet the driving mechanisms remain incompletely understood. To study the process of bifurcation, we utilized *ex vivo* three-dimensional (3D) organotypic culture coupled with time-lapse imaging to study the real-time cellular contributions to these processes (Nguyen-Ngoc et al., 2015). Our leading hypothesis for molecular regulation of bifurcation was TGF-β signaling, as it has been identified as a key regulator of side-branching and TEB migration in the mammary gland (Kahata et al., 2017; Moses and Barcellos-Hoff, 2011; Nelson et al., 2006; Silberstein and Daniel, 1987). However, our understanding of how changes in TGF-β signaling activity regulate cell behavior to accomplish bifurcation remains incomplete. Our experiments were informed by previous work using engineered devices that demonstrated that TGF-β signaling can direct cell migration by regulating the sites of cell protrusion and branch initiation (Nelson et al., 2006). We used quantitative cell tracking, analysis of cell protrusion orientation and pharmacological inhibition to study bifurcation in organotypic 3D cultures of primary murine mammary epithelium. We found that both cell speed and protrusive orientation are highly patterned in relation to nascent bifurcation points and that both are under the control of TGF-β signaling. Our work has implications for the building of organs through regenerative medicine by characterizing the rules that determine organ patterning and formation.

## RESULTS AND DISCUSSION

### Epithelial migration speed slows as cells near nascent sites of bifurcation

**Fig. 1.**
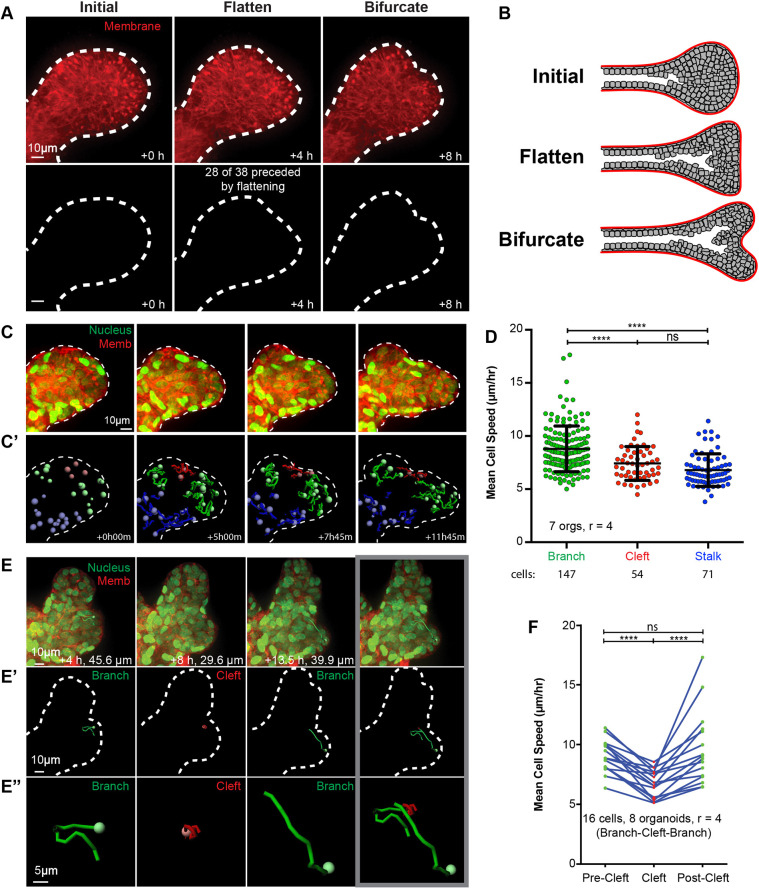
**Epithelial cell speed decreases within clefts during mammary ductal bifurcation.** (A) Confocal projection of an organoid branch undergoing bifurcation, expressing membrane-targeted tdTomato (red, top). Three representative phases of bifurcation are shown: initial, rounded; bud flattening; and cleft formation (bifurcate). In total, 28 of 38 organoids (73.7%) were observed to undergo a bud flattening phase. 38 organoids from *r*=10 replicate experiments. (B) Schematic of the three phases of mammary branch bifurcation. (C) Confocal projection of an organoid branch undergoing bifurcation expressing H2B–GFP (green) and membrane-targeted tdTomato (red). Time points are as indicated in C′. (C′) Nuclei trajectories for the organoid branch in C are shown for branch cells (green), cleft cells (red) and stalk cells (blue). Tracks represent the cell path over the previous 5 h. (D) Mean cell speeds (µm/h) were calculated from nuclei trajectories as track length divided by time for branch cells (8.79±2.15 µm/h, 147 cells), cleft cells (7.42±1.60 µm/h, 54 cells), and stalk cells (6.79±1.53 µm/h, 71 cells). Mean±s.d. of cells in seven organoids from *r*=4 replicate experiments. Kruskal–Wallis ANOVA reached significance (****P<0.0001; ns, not significant). (E) Confocal projection of an organoid branch undergoing bifurcation expressing H2B–GFP (green) and membrane-targeted tdTomato (red). Nucleus trajectories are shown for an individual cell migrating in the branch (green, +4 h) to the cleft (red, +8 h) and then returning to the branch (green, +13.5 h). Tracks represent the entirety of the path length, with path lengths for each stage indicated. Image on the right shows the complete cell track. (E′) Images showing the nucleus trajectories described in E. (E″) Magnified views of the trajectories shown in E′. (F) Paired mean cell speeds (µm/h) were calculated from nuclei trajectories as track length divided by time for individual cells that were migrating in a branch (9.03±1.36 µm/h, mean±s.d.; pre-cleft) to a cleft (6.75±1.15 µm/h) and returning to the branch (9.65±3.04 µm/h; post-cleft). Data is shown for 16 cells from eight organoids imaged in *r*=4 replicate experiments. Paired Friedman's ANOVA with Dunn's multiple comparisons reached significance (****P<0.0001; ns, not significant). Dashed lines in A,C,C′ and E′ indicate the organoid branch outline.

We began our study by imaging cell behavior during branching morphogenesis in organoids embedded within a 3D extracellular matrix (ECM) (Nguyen-Ngoc et al., 2015). Bifurcation typically occurs within 24–48 h of bud initiation, and so likely sites of bifurcation can be identified prospectively. We observed that bifurcating mammary end buds proceed through three distinct morphological stages: (1) elongation with a characteristic rounded morphology, (2) transition through a bud flattening phase and then (3) clefting to separate into two elongating buds (Fig. 1A,B). This sequence was observed in ∼75% of bifurcation events. The observed morphologies are reminiscent of the branching process in other epithelial organs (Jiang et al., 2018; Kim et al., 2015) and of morphologies observed during mammary branching *in vivo* (Silberstein, 2001a,b).

We next used quantitative cell tracking to assay for regional differences in cell behavior during branching. Bifurcating mammary end buds have three characteristic regions: the trailing stalk, the two branches, and the cleft between the branches. Using fluorescently tagged transgenic mice, we tracked individual nuclei (marked by GFP-tagged histone H2B; H2B–GFP) in each of these regions (Fig. 1C,C′). We found that cells located within branches exhibited significantly higher mean cell speed and cell persistence than cells in either the stalk or the cleft (Fig. 1D; Fig. S1B). There were no significant differences in mean cell speed or persistence between cells in the stalk or cleft.

Previous analysis of migration dynamics during elongation has revealed that epithelial cell speed and persistence are both higher in the elongation front than in the organoid body and that cells frequently exchange between regions (Huebner et al., 2016). These observations led us to hypothesize that it is the signaling environment within the tissue region that regulates cell speed, rather than pre-existing cell-autonomous differences between branch and cleft cells. To test this hypothesis, we quantified the trajectories of cells as they migrated within a branch region, approached the cleft, then exited the cleft region to enter a branch region (Fig. 1E–E″). Mean cell speed significantly decreased upon entering the cleft and returned to pre-cleft speeds upon exit (Fig. 1F; Fig. S1A). These data reveal that epithelial cell speed is differentially regulated in different regions within bifurcating ducts. Our data are consistent with cell migration analyses in branching airway epithelium (Jiang et al., 2018). Mammary epithelial cells do not appear to be pre-specified to be branch or cleft cells. Instead, they apparently respond to spatially restricted cues that determine their cell speed and eventual contribution to the pattern of the developing organ.

### Nearby mammary epithelial cells protrude anisotropically towards nascent clefts

**Fig. 2.**
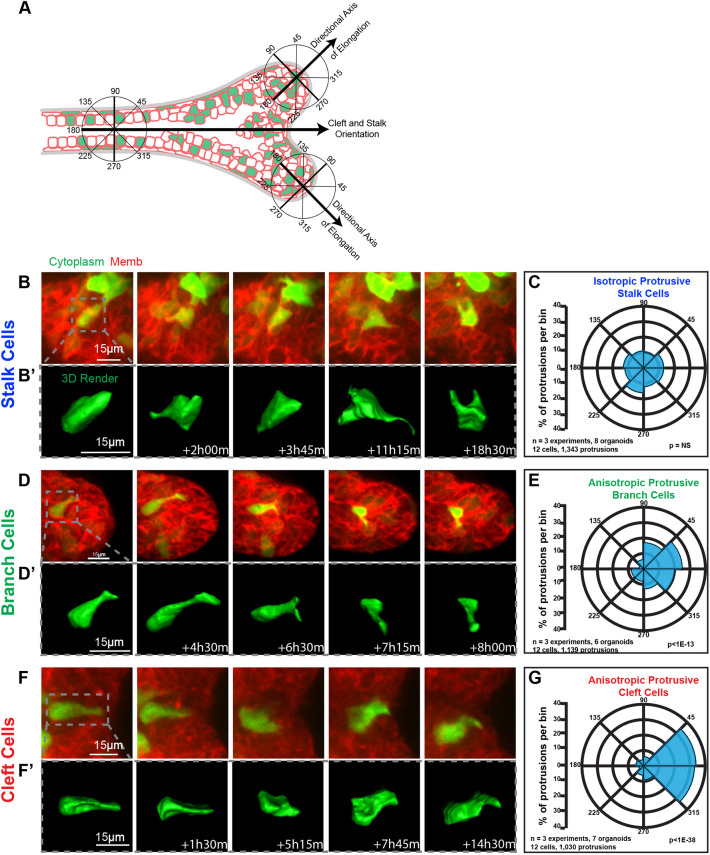
**Epithelial cells are anisotropically protrusive towards bifurcation clefts.** (A) Schematic illustrating overlay method and orientation for assigning protrusions to 45° bins. Branch cells are aligned with the direction of elongation. Stalk and cleft cells are aligned with the bisection line between the two elongating branches. (B,B′) A representative confocal projection (B) and 3D reconstruction (B′) of a stalk cell with isotropic protrusions, imaged during active bifurcation. (C) Polar histogram showing protrusions per bin quantified from organoid stalk cells (1343 protrusions from 12 cells in eight organoids imaged in *r*=3 replicate experiments). Two-way ANOVA did not reach significance (NS, *P*>0.05). (D,D′) A representative confocal projection (D) and 3D reconstruction (D′) of a branch cell with anisotropic protrusions, imaged during active bifurcation. (E) Polar histogram showing protrusions per bin quantified from organoid branch cells (1139 protrusions from 12 cells in six organoids imaged in *r*=3 replicate experiments). Two-way ANOVA reached significance (*P*<1×10^−13^). Two-way MANOVA reached significance for the comparison between stalk and branch cells (*P*<5×10^−8^). (F,F′) A representative confocal projection (F) and 3D reconstruction (F′) of a cleft cell with anisotropic protrusions, imaged during active bifurcation. (G) Polar histogram showing protrusions per bin quantified from organoid cleft cells (1030 protrusions from 12 cells in seven organoids imaged in *r*=3 replicate experiments). Two-way ANOVA reached significance (*P*<1×10^−38^). Two-way MANOVA reached significance for the comparison between stalk and cleft cells (*P*<1×10^−10^). Branch cells were not compared to the cleft cells as they have different axes of orientation. Cells in B,D and F are labeled with H2B–GFP (green) and membrane-targeted tdTomato (red).

Mammary epithelial cells are highly protrusive and migratory during branching morphogenesis, though these protrusions do not extend into the surrounding ECM (Ewald et al., 2008, 2012; Neumann et al., 2018). Mammary epithelial protrusions are patterned in relationship to tissue growth, as cells within the bud are selectively protrusive in the direction of elongation (anisotropic), whereas cells in the body of an organoid extend protrusions with equal probability in all directions (isotropic) (Huebner et al., 2016). In the present study, we sought to determine how the direction of protrusions changed as an end bud bifurcated. We quantified the orientation of protrusions using a sector chart for the three regions: stalk, elongating branches and clefts (Fig. 2A). Each chart was divided into eight equally spaced sectors of 45°. For the branch sector charts, 0° was aligned in the direction of branch elongation. The location where the orientation axes of the two branch sector charts intersected was then bisected towards the cleft to give the 0° orientation used for the cleft and stalk sector charts. Protrusions located within the 0–45° and 315–360° bins were considered as being in the direction of either branch elongation or the cleft. Protrusions that were placed within the 135–225° bins were considered as opposing the direction of branch elongation or the cleft.

We observed that cells within the stalk were isotropically protrusive, showing no mean direction (Fig. 2B–C). In contrast, cells within branches undergoing bifurcation were anisotropically protrusive in the direction of branch elongation (Fig. 2D–E), consistent with previous work from our lab (Huebner et al., 2016). Furthermore, we found that the protrusions of cells near the cleft were highly anisotropic towards the cleft (Fig. 2F–G). Clefting in other epithelial systems requires acto-myosin contractility to form the cleft and bifurcate (Andrew and Ewald, 2010; Kim et al., 2015; Wang et al., 2017). It is unclear at present the extent to which similar mechanisms regulate mammary bifurcation, as organoids in our assay are still capable of some branching when treated with myosin light chain kinase inhibitor (ML7) or Rho kinase (ROCK) inhibitor (Y27632) (Ewald et al., 2008).

### TGF-β signaling regulates mammary branching pattern

**Fig. 3.**
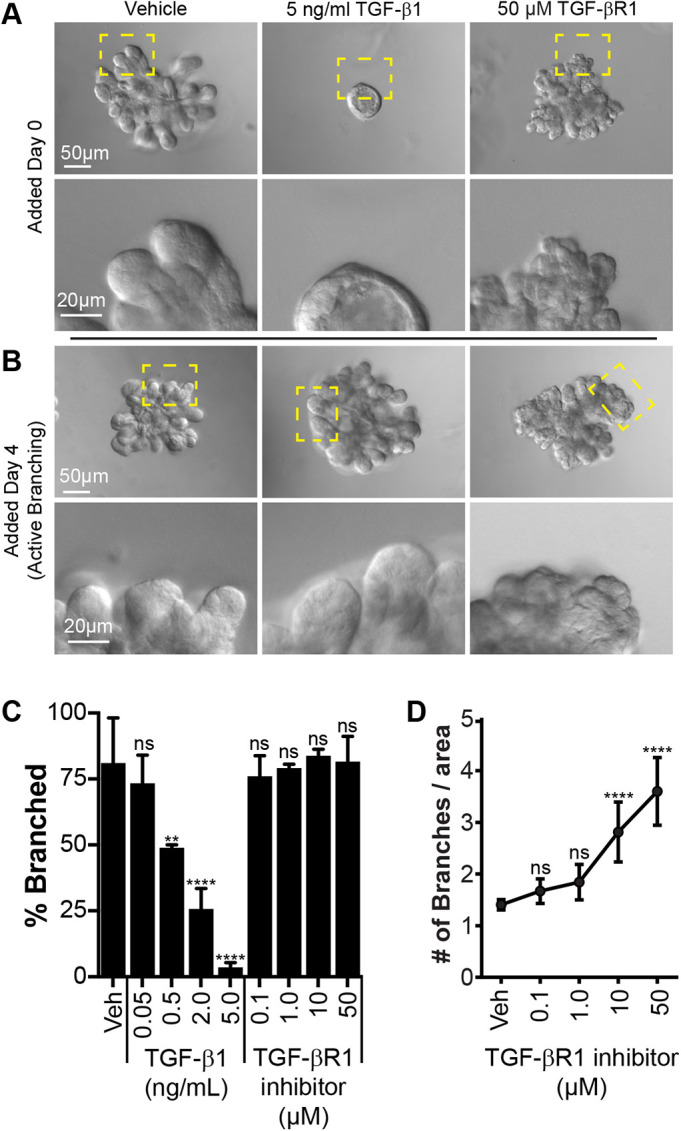
**Inhibition of TGF-β signaling results in hyperbranching of organoids.** (A) DIC images taken at day 7 showing organoids that were cultured from day 0 in the presence of vehicle (DMSO), 5 ng/ml exogenous TGF-β1 or 50 µM TGF-βR1 inhibitor (LY364947). Yellow boxes indicate regions shown in magnified images (bottom). (B) DIC images taken at day 7 showing organoids that were cultured from day 4 (active branch elongation) in the presence of vehicle (DMSO), 5 ng/ml exogenous TGF-β1 or 50 µM TGF-βR1 inhibitor (LY364947). Yellow boxes indicate regions shown in magnified images (bottom). (C) Mean±s.d. percentage of organoids forming branches at day 7 following treatment with the indicated concentrations TGF-β1, TGF-βR1 inhibitor or vehicle (Veh; DMSO) from day 0. Vehicle, 81.3±17.1% (269 organoids). Exogenous TGF-β1: 0.05 ng/ml, 73.4±10.6% (368 organoids); 0.5 ng/ml, 49.0±1.1% (310 organoids); 2.0 ng/ml, 25.7±7.7% (386 organoids); 5.0 ng/ml, 3.6±1.7% (274 organoids). TGF-βR1 inhibitor (LY364947): 0.1 µM, 76.0±7.8% (426 organoids); 1.0 µM, 79.1±1.5% (399 organoids); 10 µM, 83.9±2.5% (398 organoids); 50 µM, 81.6±9.6% (409 organoids). Data are from *r*=3 replicate experiments. Ordinary two-way ANOVA with Tukey's multiple comparison's test reached significance (ns, *P*>0.05; **P<0.01; ****P<0.0001). (D) Mean±s.d. number of branches per area for organoids at day 7 following treatment with vehicle (Veh; DMSO) or the indicated concentration of TGF-βR1 inhibitor (LY364947) from day 0. Vehicle, 1.41±0.10; 0.1 µM, 1.67±0.24; 1 µM, 1.84±0.34; 10 µM, 2.81±0.58; and 50 µM, 3.60±0.66. Data are from 10 organoids per condition, *r*=3 replicate experiments. Ordinary one-way ANOVA with Holm–Sidak multiple comparison test with a single-pooled variable reached significance (ns, *P*>0.05; ****P<0.0001).

TGF-β is established as an *in vivo* regulator of mammary branching morphogenesis, yet the cellular basis of its effects remains incompletely understood (Ewan et al., 2002; Moses and Barcellos-Hoff, 2011; Nelson et al., 2006). Accordingly, we tested the effects of perturbations of TGF-β signaling on branching morphogenesis in our 3D organotypic cultures. We found that early treatment with exogenous TGF-β1 (TGFB1) resulted in a dose-dependent abrogation of branching morphogenesis (Fig. 3A,C). This is consistent with previous reports on the role of TGF-β1 as a concentration-dependent growth inhibitor (Daniel et al., 1989; Nelson et al., 2006; Pavlovich et al., 2011; Pierce et al., 1993; Silberstein and Daniel, 1987). For example, treatment of TEBs with TGF-β1-coated beads during active elongation *in vivo* leads to regression of these buds, which occurs in a reversible fashion (Silberstein and Daniel, 1987). We hypothesized that treatment with TGF-β1 during active elongation (day 4 in culture) would also lead to regression of branches. However, late treatment with TGF-β1 had no effect on the percentage of buds that formed branches, nor did it lead to obvious differences in branch morphology (Fig. 3B), which is consistent with the results of studies overexpressing TGF-β in mature ducts *in vivo* prior to alveologenesis (Jhappan et al., 1993). These results together suggest a nuanced role for TGF-β signaling during morphogenesis, with effects being modulated by various cell populations and ECM interactions (Moses and Barcellos-Hoff, 2011). Interestingly, we have previously shown that proliferation is required early in these cultures to generate a low-polarity stratified epithelial tissue architecture that is a necessary precursor to branching. In contrast, if proliferation is inhibited after the multilayered epithelium has formed (but prior to budding), organoids can still undergo branching, indicating that proliferation has stage-specific roles in branching morphogenesis (Huebner et al., 2016).

We next blocked TGF-β signaling using a TGF-βR1 (TGFBR1) inhibitor (LY364947). We found that both early and late inhibition of TGF-β signaling led to a hyper-branched morphology, although a similar overall number of branched organoids was retained following inhibitor treatment (Fig. 3A–D). Compared to control organoids, the inhibitor-treated organoids had more clefts and their branches were shorter and appeared stunted. Work from other groups has shown that TGF-β1 heterozygous mice display more rapid growth and increased branching in the mammary gland (Ewan et al., 2002). Another group has shown that expression of a dominant-negative TGF-β receptor specifically in the mammary stroma leads to an increase in epithelial branching, which is consistent with our inhibition data, suggesting the importance of tissue–ECM interactions (Joseph et al., 1999).

### Epithelial cell speed is regulated by TGF-β signaling

**Fig. 4.**
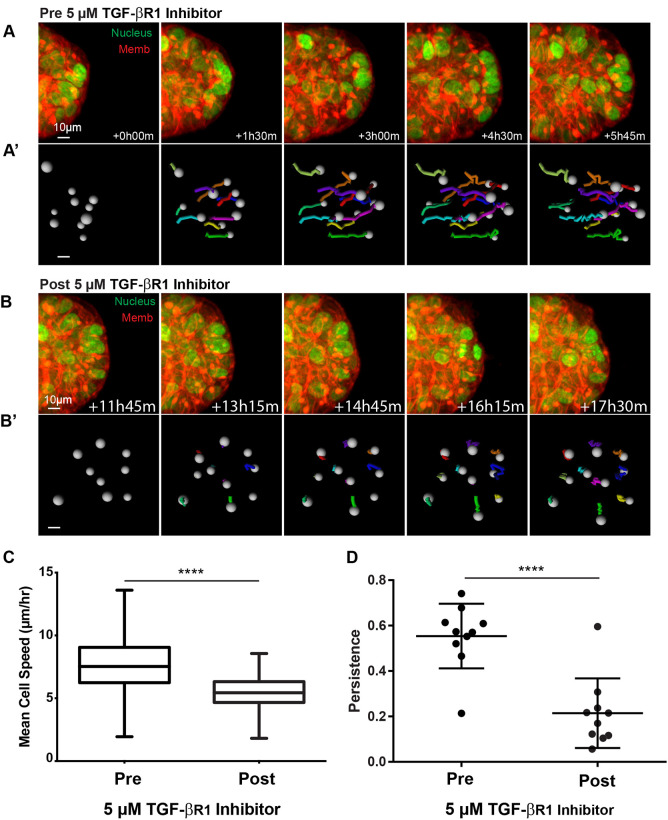
**TGF-β1 signaling is acutely required for epithelial cell migration.** (A) Confocal projections of an organoid branch undergoing elongation, expressing H2B–GFP (green) and membrane-targeted tdTomato (red). (A′) Nuclei trajectories for the branch shown in A, with tracks in multiple colors to allow identification at different time points. Tracks represent the cell path over the previous 5 h. Scale bar: 10 μm. (B) Confocal projections showing the same organoid branch as depicted in A following treatment with 5 µM TGF-βR1 inhibitor (LY364947). Times are shown relative to the start of imaging pre-inhibition. (B′) Nuclei trajectories from the branch shown in B, with tracks in multiple colors to allow identification at different time points. The last 6 h of imaging are displayed. Tracks represent the cell path over the previous 5 h. Scale bar: 10 μm. (C) Mean cell speeds (µm/h) in organoid branches were calculated from nuclei trajectories as track length divided by time for cells before (Pre; median, 6.55 µm/h; interquartile range, 7.72–9.17 µm/h; 2535 cells) and after (Post; median, 4.66 µm/h; interquartile range, 5.44–6.32 µm/h; 3821 cells) treatment with 5 µM TGF-βR1 inhibitor (LY364947). Cells were from 69 organoids imaged in *r*=4 replicate experiments. Two-tailed unpaired *t*-test reached significance (****P<0.0001). Boxplots show median and interquartile range, with whiskers marking the minimum and maximum cell speeds. (D) Persistence was calculated from nuclei trajectories as displacement length divided by total track length for cells in organoid branches before (0.55±0.14, 10 cells) and after (0.21±0.15, 10 cells) treatment with 5 µM TGF-βR1 inhibitor (LY364947). Mean±s.d. of cells from four organoids imaged in *r*=3 replicate experiments. Two-tailed unpaired *t*-test reached significance (****P<0.0001).

TGF-β signaling has been extensively characterized during morphogenesis of branched epithelial organs (Kahata et al., 2017); however, its role in regulating cell migration during epithelial morphogenesis remains incompletely understood. Previous analysis has revealed that TGF-β1-null mice have decreased overall TEB length, similar to the effect of TGF-βR1 inhibition in our organoids (Ingman and Robertson, 2008). These data led us to hypothesize that TGF-β signaling regulates branching morphogenesis through effects on cell migration. Using fluorescently tagged transgenic mice, we tracked individual nuclei (marked by H2B–GFP) during active elongation of mammary ducts in 3D organotypic culture prior to and after treatment with TGF-βR1 inhibitor (Fig. 4A–B′). We found that inhibition of TGF-β signaling led to acute reductions in cell migration speed and persistence (Fig. 4C,D). The magnitude of reduction in speed and persistence induced by inhibition of TGF-β signaling is similar to that observed when cells enter a cleft region during spontaneous bifurcation (Fig. 1F). We therefore speculate that endogenous branching is regulated by spatially patterned TGF-β signaling acting, at least in part, through regulation of cell migration.

A limitation of our study is that the addition of soluble TGF-βR1 inhibitor likely affects all cells in the culture. Our cell tracking suggests that cell migration speed is reduced broadly, and yet we see an increase in branching. We speculate that this hyper-branching occurs because TGF-β1 inhibitor differentially affects cleft cells and branch cells. Our working hypothesis is that branching requires an ‘escape velocity’ of branch cells relative to cleft cells. The global decrease in cell speed from TGF-β1 inhibition makes it ‘easier’ for branch cells to achieve this escape velocity, thus creating more clefts and branches, and therefore a hyperbranched tissue-level phenotype. Put another way, the mean cell speed ratio of branch cells compared to cleft cells is greater upon inhibition of TGF-β1. For example, in the control condition we observe migration speeds of ∼10 μm/h in branch cells versus ∼8 μm/h in cleft cells, thus branch cells have a migration speed that is 1.25 times that of cleft cells. Upon TGF-βR1 inhibition, the migration speeds of branch cells are closer to ∼6 μm/h, and cleft cells might migrate at closer to ∼4 μm/h, thus branch cells migrate at 1.5 times the speed of cleft cells. That difference in ratio of 1.25 versus 1.5 could result in more branching events.

Future work will need to investigate how the mammary gland coordinates heterotypic intercellular interactions while transducing and interpreting TGF-β, RTK and ECM signals to regulate branching morphogenesis. This interplay is likely to be complex and reciprocal, since TGF-β signaling has been shown to regulate ECM deposition during morphogenesis (Silberstein and Daniel, 1982; Silberstein et al., 1992; Verrecchia and Mauviel, 2002). Additionally, integrin binding and signaling are key regulators of myoepithelium–ECM interactions during morphogenesis (Nisticò et al., 2014; Schedin and Keely, 2011). Our work is also consistent with the recent demonstration that ECM accumulation at the cleft along the flanks of TEBs plays a key role in mammary bifurcation (Nerger et al., 2021). Future work should also investigate how different ECM components signal through integrins to regulate interactions between myoepithelial and luminal epithelial cells during branching. It will also be important to determine how the individual cell migration behaviors documented in this study interact with underlying biophysical mechanisms, such as tissue bending and cell shape change.

## MATERIALS AND METHODS

### Transgenic animals

A dual-transgenic mouse line expressing a membrane label, ROSA26:: tdTomato [Jackson Laboratory, stock #007676; ROSA26Sor^tm4(ACTB-tdTomato,-EGFP)Luo^], and a nuclear label, CAG::H2B–GFP [kind gift from A. K. Hadjantonakis, Memorial Sloan Kettering Cancer Center, NY, USA; Jackson Laboratory, stock #006069; Tg(HIST1H2BB/EGFP)1Pa; Hadjantonakis and Papaioannou, 2004] was utilized for time-lapse imaging. Wild-type FVB/NJ mice (Jackson Laboratory, stock #00180) were utilized in assays. Animal experiments were conducted in accordance with protocols approved by JHU Medicine Institutional Animal Care and Use Committee.

### 3D organotypic culture

The 3D *ex vivo* organotypic culture methods and their use have been described in detail previously (Ewald, 2013a; Ewald et al., 2008; Nguyen-Ngoc et al., 2015). Briefly, mouse mammary glands from 8–12-week-old female mice were dissected. After mincing with a scalpel, the isolated glands were treated with collagenase–trypsin, DNase, and differential centrifugation to separate the fat and stromal tissue from the epithelium. Organoids were then embedded in a 1:1 mixture of growth factor reduced Matrigel (BD Biosciences) and fibrillar rat tail collagen I (Corning), plated and polymerized on 24-well glass-bottom plates (Greiner Bio One) as 150 µl gels (1–2 organoids/µl) at 37°C. Branching morphogenesis was stimulated using 2.5 nM FGF2 (F0291, Sigma-Aldrich). Statistical analysis and measurements were performed either using organoids or individual cells within organoids, and organoids were randomly assigned to conditions when applicable. For branching assays, branched organoids were defined as having three or more branches; unbranched organoids were defined as having fewer than three branches.

### Adenoviral infection

After organoid isolation, but prior to suspension in ECM, the 1:1 Matrigel-fibrillar collagen I matrix described above, adenoviral eGFP (Vector Biolabs) was added, as described previously (Huebner et al., 2016). Isolated organoids were centrifuged, resuspended in 100 µl of DMEM-F12 (Gibco), and adenovirus added at 10,000 plaque-forming units per organoid to achieve gene expression in ∼50% of cells. Organoids were incubated with virus for 1 h at 37°C and then washed twice with DMEM-F12 and suspended in ECM for plating. Adenoviral eGFP infection has been previously shown to have no effect on cell migration speeds (Neumann et al., 2018).

### Confocal microscopy

Time-lapse images were acquired using a spinning disk confocal microscope (Solamere Technology Group) and a 40× LD-LCl C-Apochromat lens (Ewald, 2013b). The spinning disk microscope used microManager64 (https://micro-manager.org/) and Piper (Stanford Photonics) to acquire images. Organoids were imaged for a duration of 12 h to 24 h with a 10–15 min frame interval, with the temperature maintained at 37°C and CO_2_ at 5%. Brightness and contrast were adjusted across the entire image using Imaris (Bitplane) to maximize image clarity.

### Differential interference contrast microscopy

Using the Zeiss Cell Observer, with an AxioObserver Z1 and an AxiocamMRM camera, differential interference contrast (DIC) images were collected of organoids at day 7 that were fixed in 4% paraformaldehyde for 10 min and washed three times in PBS (Ewald, 2013b).

### Nuclei tracking analysis

Using Imaris (Bitplane), individual nuclei were tracked using the Spots function. Within a reconstituted 3D image, the centers of distinguishable nuclei were marked. Within each time frame, spots were either connected by the software or manually. Spots were then confirmed manually in either the Surpass view mode or Imaris OrthoSlicer function to ensure accurate placement of marks on nuclei. Mean cell speeds were quantified as total track length divided by duration of tracking. Persistence was calculated from nuclei trajectories as displacement length divided by total track length. When possible, analyses were blinded or automated. Datasets were batch processed within Imaris (Bitplane) after the initial parameters were identified to ensure data robustness and then applied consistently to the data across groups.

### Cellular protrusion analysis

Using the organotypic culture assay and adenoviral gene delivery, the cytoplasm of a mosaic subset of cells was labeled with eGFP. After time-lapse imaging on the confocal microscope (every 10–15 min for 12–24 h) and reconstitution of the 3D images using Imaris (Bitplane), cellular protrusions of cells with cytoplasmic expression of eGFP were analyzed using the Surfaces function to reconstruct a 3D model of GFP-expressing cells. Protrusions were manually analyzed using an eight-section pie with deviations every 45° on a transparent sheet. The axis of the pie was aligned with either the direction of branch elongation or the bisector of the two elongating branches during bifurcation to mark the cleft. The number of protrusions in each bin for each cell were counted for a minimum of 10 h. Protrusion data were plotted as a polar histogram using MATLAB (MathWorks), using the mean values for each bin. Statistical analyses were performed in MATLAB. Analysis of variance (ANOVA) was used to determine the significance of a weighted mean direction. The null hypothesis is that there is no mean direction. Multivariate analysis of variance (MANOVA) was used to determine the significance of the difference in the weighted mean direction between datasets. The null hypothesis is that there is no difference in the mean direction. Organoids were included in the analysis if the organoid was undergoing bifurcation. When possible, analyses were blinded; however, it was not possible to prevent an individual from identifying the location of a cell in an elongating branch versus a cleft, as these locations occur in the same time-lapse dataset. Bias was reduced by showing the raw dataset to multiple individuals and having multiple individuals independently analyze a portion of the datasets.

### Molecular perturbation assays

TGF-β signaling was modulated using either TGF-β receptor type-1 inhibitor (LY-364947; Tocris, #2718) at 0.1, 1, 5, 10 or 50 µM, with DMSO used as a vehicle control, or exogenous TGF-β1 (Sigma, #T7039) at 0.05, 0.5, 2.0 or 5.0 ng/ml. Treatments were added on day 0 or day 4 in culture, as indicated. To assess the degree of branching, organoids were incubated at 37°C with their respective concentrations of inhibitor or exogenous TGF-β1 and fixed at day 7. DIC images of organoids were then acquired. To assess the effects of inhibition of TGF-β signaling on cell speed, elongating organoids expressing tdTomato and H2B–GFP were imaged for 6 h prior to inhibition and for 12–16 h following inhibition. Mean speeds were quantified, and a two-tailed *t*-test was performed for statistical analysis. Each experiment was set up in such a way that the DIC images were acquired and analysed solely based on well location. Once analyzed, the well-plate locations were retroactively correlated with the perturbation condition.

## Supplementary Material

10.1242/joces.259275_sup1 Supplementary information
