# Supplementary material for: Collective cell migration is spatiotemporally regulated during mammary epithelial bifurcation
Source: J Cell Sci. 2023 Jan 5;136(1):jcs259275. doi: 10.1242/jcs.259275 (PMC10112963; doi:10.1242/jcs.259275)
Supplement: Supplementary information [file joces-136-259275-s1.pdf]

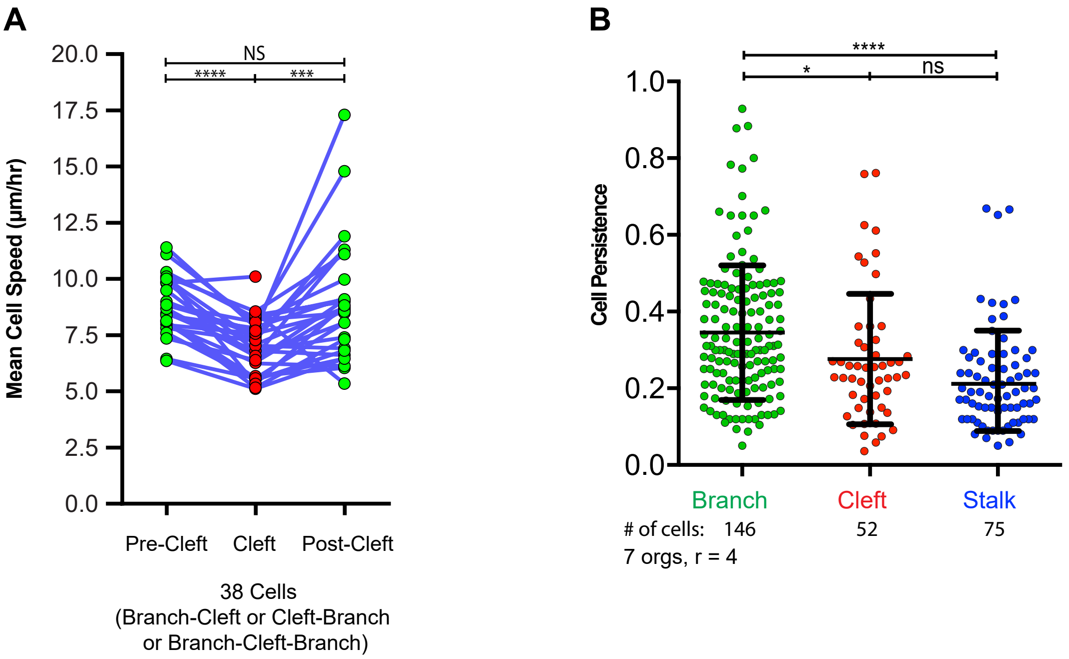

Fig. S1. Migratory epithelial cells display spatially-dependent effects on speed within the bifurcation clefts.

(A) Paired mean cell speeds (µm/hour) were calculated from nuclei trajectories as track length divided by time for individual cells that were migrating between all three locations: a branch (8.77 ± 1.26 µm/hr, pre-cleft), to a cleft (7.00 ± 1.17 µm/hr), and returned to the branch (8.89 ± 2.67 µm/hr, post-cleft), or did two of the three locations (38 cells from 10 organoids, r = 7). Quantification includes the data from Figure 1F. Paired analysis ANOVA reached significance (****$p < 0.0001$). (B) Cellular persistence (path length divided by displacement length) was measured for branch (0.35 +/- 0.18), cleft (0.28 +/- 0.17), and stalk (0.22 +/- 0.13) cells. Paired analysis ANOVA reached significance (*$p<0.05$; ****$p < 0.0001$). Mean ± SD, 7 organoids, r = 4.

Journal of Cell Science • Supplementary information
